# Effect of zinc level and the thermal environment on the zootechnical performance and tibia-breaking strength of Japanese quails

**DOI:** 10.3389/fvets.2024.1467487

**Published:** 2025-01-08

**Authors:** Luiz Arthur dos Anjos Lima, Thiago de Assis Moraes, Larissa Kellen da Cunha Morais, Mikael Leal Cabral Menezes de Amorim, Tarsys Noan Silva Veríssimo, José Danrley Cavalcante dos Santos, Maria Isabelly Leite Maia, Severino Guilherme Caetano Gonçalves dos Santos, Fernando Guilherme Perazzo da Costa, Ricardo Romão Guerra, Lucas Rannier Ribeiro Antonino Carvalho, Edilson Paes Saraiva

**Affiliations:** ^1^Research Group in Bioclimatology, Ethology and Animal Welfare (BioEt), Department of Animal Science, Federal University of Paraiba, Areia, Paraiba, Brazil; ^2^Graduate Program in Animal Science and Fisheries Resources, Faculty of Agricultural Sciences, Federal University of Amazonas, Manaus, Amazonas, Brazil; ^3^Department of Animal Science, Federal University of Paraiba, Areia, Paraiba, Brazil; ^4^Department of Veterinary Sciences, Federal University of Paraiba (UFPB), Areia, Paraiba, Brazil; ^5^Department of Physiology and Pharmacology, Karolinska Institutet, Stockholm, Sweden

**Keywords:** animal nutrition, *Coturnix coturnix japonica*, heat stress, mineral supplementation, weight gain

## Abstract

Japanese quails (*Coturnix coturnix japonica*) are sensitive to zinc (Zn) deficiency, a mineral essential for growth, development, and bone health. This study evaluated the effects of different levels of Zn in the diet on zootechnical performance, organ and carcass weight, and tibial breakage resistance in quails from 1 to 42 days of age. A 5 × 2 factorial design was used, consisting of five Zn levels (30, 60, 90, 120, and 150 mg/kg) and two thermal environments (thermal comfort and heat stress), with five replicates of 10 birds per treatment. The results indicate an antagonistic relationship between high levels of Zn in the diet (150 mg/kg) and quail performance, where the addition of the highest Zn level impaired performance. On the other hand, supplementation with the available Zn from the basal diet (30 mg/kg) was sufficient to ensure satisfactory weight gain, better feed conversion, and appropriate carcass and liver weights of quails during the initial rearing phase. At 42 days, supplementation with 150 mg/kg of Zn provided greater resistance to tibial breakage, regardless of thermal conditions. These findings highlight the importance of adjusting Zn supplementation according to the needs of quails at different rearing stages. The study emphasizes the need for a balanced nutritional approach, considering both adequate Zn levels and the management of thermal stress. The combination of appropriate Zn levels in the diet and environmental management, especially concerning thermal stress, is crucial to optimizing productive performance, bone health, and the well-being of the birds. Furthermore, the nutritional Zn requirements for quails in the initial rearing phase may be lower than previously established, without compromising performance. Gradual Zn supplementation, according to the needs of the production stage and environmental conditions, is essential to ensure the health and productivity of the birds.

## 1 Introduction

The intensification of climate change over the years, evidenced by the continuous rise in global temperatures, has highlighted environmental factors as one of the main challenges in animal production systems. The rearing environment plays a crucial role in animal productivity and welfare, but it often fails to meet their optimal physiological needs (1). This mismatch results in adverse consequences, such as reduced feed intake, lower growth rates, impaired development, and decreased feed conversion efficiency. These impacts are particularly pronounced in intensive poultry production systems, where high ambient temperatures negatively affect the birds’ physiology, immunity, and, consequently, productivity (2).

In this context, integrated approaches that combine environmental management strategies and nutritional adjustments have gained prominence as promising tools to mitigate the effects of heat stress (3). Environmental modifications, including proper ventilation, shading, and cooling systems, are essential to alleviate the impact of adverse thermal conditions. Additionally, nutritional interventions, particularly through supplementation with essential minerals such as zinc (Zn), can enhance the animals’ adaptive capacity. Zinc plays critical roles as a cofactor in various metabolic reactions, including cell division, hormonal synthesis, and enzymatic activity (4, 5). Moreover, its anti-stress properties are widely recognized, being associated with the maintenance of immunity and metabolism under unfavorable environmental conditions (6).

Although significant advances have been made in the use of minerals in animal nutrition, the interactions between zinc supplementation and thermal conditions remain underexplored, particularly in Japanese quails. This species is known for its high sensitivity to zinc deficiency, which is indispensable for adequate growth, tibia conformation and length, and overall performance. Previous studies suggest that dietary zinc requirements for quails, both during growth and adulthood, range from 25 mg/kg to 50 mg/kg of feed (7). However, significant gaps persist in understanding how this mineral influences the metabolism and performance of quails reared under high ambient temperatures, emphasizing the need for further research on this topic.

Based on this context, the present study aimed to address the following research questions: (1) How do different levels of dietary zinc supplementation affect the zootechnical performance, organ and carcass weights, and bone strength of Japanese quails raised under normal and heat-stressed thermal conditions? and (2) Is there a significant interaction between zinc levels and thermal environment that modulates the birds’ responses? The tested hypothesis was that zinc supplementation at levels exceeding traditional requirements could mitigate the adverse effects of heat stress, improving the zootechnical performance, bone strength, and carcass quality of Japanese quails. Thus, this study aims to contribute to the understanding of the interactions between nutrition and the rearing environment, providing scientific evidence to support more effective management practices, particularly in hot climate regions.

## 2 Methods

### 2.1 Experiment location, birds, treatments and diets

This research was approved by the Animal Use Ethics Committee (CEUA) of the Federal University of Paraiba (Protocol No. 072/2016). The experiment was conducted in climatic chambers to the Bioclimatology, Ethology and Animal Welfare Research Unit of the Agrarian Sciences Center, Federal University of Paraiba, located in Areia, Paraiba, Brazil.

A total of 500 Japanese quails female (*Coturnix coturnix japonica*), 1 day old, with an initial weight of 7.0 g ± 0.5 g, were distributed in a completely randomized experimental design in a 5 × 2 factorial design, with diets containing five levels of Zn (30, 60, 90, 120 and 150 mg/kg of feed), five replicates in standard wire cages (10 birds per cage, measured at 55 × 55 cm) in two climate chambers with environmental control, thermal comfort and heat stress (25 cages per room).

Each climatic chamber contained an area of 19.71 m^2^, equipped with air conditioning, heater, humidifier, dehumidifier, exhaust fans and thermostat, monitored by a computerized system located in a control room. The boxes were equipped with pacifier-type feeders and drinkers.

As the thermal needs of domestic birds change according to their growth, based on previous works (4), different thermal conditions were defined for each of the climatic chambers (Table 1). The average relative humidity of the air inside the two chambers varied between 65 and 70%.

**Table 1:** Temperature used in the climatic chambers for two environmental conditions according to the age of birds.

Age (days)	Thermal comfort (°C)	Heat stress (°C)
1 to 7	36	41
8 to 14	32	37
15 to 21	28	34
22 to 42	26	33

All quail were vaccinated against Newcastle and Gumboro disease via water and were under a light program of 24 h from the first to the 20th day, 18 h from day 21–34 and 17 h from day 35–42.

The chemical composition of the ingredients used in the formulation of the experimental diets was determined through near infrared spectroscopy (NIRS). The experimental diets were formulated with corn and soybean meal according to the nutritional recommendations suggested by Rostagno et al. (5) (Table 2).

**Table 2:** Composition and nutrient content of experimental diets for Japanese quail aged 1 to 42 days old.

Ingredients (kg)	Zn levels (mg kg^−1^)				
	30	60	90	120	150
Corn grain, 7.88%	56.211	56.211	56.211	56.211	56.211
Soybean bran, 45%	38.518	38.518	38.518	38.518	38.518
Soybean oil	1.373	1.373	1.373	1.373	1.373
Calcitic limestone	1.196	1.196	1.196	1.196	1.196
Dicalcium phosphate	1.387	1.387	1.387	1.387	1.387
Common salt	0.406	0.406	0.406	0.406	0.406
DL-methionine	0.165	0.165	0.165	0.165	0.165
L-lysine HCl	0.030	0.030	0.030	0.030	0.030
L-threonine	0.037	0.037	0.037	0.037	0.037
Choline chloride, 60%	0.070	0.070	0.070	0.070	0.070
Mineral premix (Zn-free)^1^	0.100	0.100	0.100	0.100	0.100
Vitamin premix^2^	0.100	0.100	0.100	0.100	0.100
Coccidiostat (Coxistac)^3^	0.050	0.050	0.050	0.050	0.050
Growth promoter (Surmax)^4^	0.005	0.005	0.005	0.005	0.005
Butyl-hydroxy-toluene (BHT)	0.010	0.010	0.010	0.010	0.010
Zn sulfate, 35%	0.000	0.086	0.171	0.257	0.342
Inert	0.342	0.256	0.171	0.085	0.000
Total	100	100	100	100	100
Chemical composition					
Crude protein (%)	22.00	22.00	22.00	22.00	22.00
Metabolizable energy (kcal kg^−1^)	2,900	2,900	2,900	2,900	2,900
Methionine + digestible cysteine (%)	0.760	0.760	0.760	0.760	0.760
Digestible lysine (%)	1.120	1.120	1.120	1.120	1.120
Digestible threonine (%)	0.790	0.790	0.790	0.790	0.790
Digestible valine (%)	0.940	0.940	0.940	0.940	0.940
Tryptophan (%)	0.250	0.250	0.250	0.250	0.250
Calcium (%)	0.900	0.900	0.900	0.900	0.900
Available phosphorus (%)	0.375	0.375	0.375	0.375	0.375
Sodium (%)	0.180	0.180	0.180	0.180	0.180
Chlorine (%)	0.300	0.300	0.300	0.300	0.300
Potassium (%)	0.860	0.860	0.860	0.860	0.860
Zinc (mg kg^−1^)	30	60	90	120	150

1 Mineral premix (per kg): 10 mg of Cu, 50 mg of Fe, 80 mg of Mn, 1.2 g of I.

2 Vitamin premix (per kg): 8,000 IU of vitamin A, 2,000 IU of vitamin D3, 15 mg of vitamin E, 2 mg of vitamin K, 3 mg of vitamin B1, 4 mg of vitamin B2, 2 mg of vitamin B6, 10 mg of vitamin B12, 60 mg of biotin, 15 mg of pantothenic acid, 30 g of niacin, 7 mg of folic acid, 4 g of selenium.

3 Salinomycin 12%, phibro.

4 Avilamycin.

According to Hussein and AL-Bayar (6), the dietary zinc (Zn) requirements for growing and adult Japanese quails range from 25 mg/kg to 50 mg/kg of feed. Based on this recommendation, a basal diet was formulated using a zinc-free mineral premix. The other diets were prepared with increasing levels of Zn, with zinc sulfate (ZnSO_4_), having 35% bioavailability, added to replace the inert component in the diet to achieve the desired zinc levels.

Food and water were provided *ad libitum* to the animals, and for this purpose, the feeders were filled twice a day (7:00 and 19:00).

### 2.2 Zootechnical performance

On days 7, 14, 21, 35, and 42 of confinement, all the birds of de experimental unit and the remaining feed for each plot were weighed to determine weight gain (WG), feed intake (FI) and de feed conversion ratio (FCR). The WG was determined by the difference between the final and initial weights; the FI, by the difference between the feed provided and the remaining feed collected; and FCR, by the relationship between FI and WG.

### 2.3 Body and carcass weight

To evaluate body weight and carcass, on days 22 and 42 of feedlot, two quails were randomly collected from each experimental unit, totaling 100 birds per period, identified and weighed. Each quail was euthanized by cervical displacement, plucked and eviscerated to obtain the weight of the carcass. The internal organs (spleen, liver, and cloacal pouch) were also separated. All organs were weighed individually on a precision scale to calculate the relative weight (%); relative weight was calculated as organ weight/bird weight × 100.

### 2.4 Tibia breaking strength

At 42 days of age, samples were collected from the left tibiae of two birds per experimental unit, chosen at random, for analysis of resistance to bone flexion (7). After drying at room temperature, the tibias were placed in a TA-XT Plus universal testing machine (Stable Micro Systems, Surrey, United Kingdom) which records the flexural strength of solid materials (8). The brackets were designed in the horizontal position and the two were applied in the central position. The maximum amount of force (kgf) applied to the bones at the moment of rupture was considered as flexural strength.

### 2.5 Data analysis

The performance variables, organ and carcass weight, and tibial rupture force were submitted to analysis of variance (ANOVA) using the SAS software (9). The level of statistical significance was set at *p* < 0.05, and the best level of Zn supplementation was estimated using polynomial regression models.

Tukey’s test compared the difference between dietary zinc levels, while the *t*-test compared the mean environmental effects.

When adjusted to a quadratic regression, the equations were derived by estimating the maximum and minimum levels of Zn supplementation (10).

## 3 Results

### 3.1 Zootechnical performance

Interaction effects between Zn levels and the thermal environment on the performance parameters were not observed in the 1 to 21-day phase (Table 3). There was effect of dietary Zn levels on quail WG (*p* = 0.0386) and FCR (*p* = 0.0060). Regression analysis revealed that WG decreased linearly (*p* = 0.0078), whereas FCR increased linearly (*p* = 0.0007) with an increase in Zn levels in the diet (Table 3).

**Table 3:** Performance of Japanese quail aged 1 to 21 days old, supplemented with zinc levels in the diet and reared under different thermal environments (*n* = 500).

Zn levels (mg kg^−1^)	*n*	Feed intake (g bird^−1^)	Weight gain (g bird^−1^)	Feed conversion ratio (g/g/bird)
30	100	197.02	86.84ab	2.27b
60	100	196.84	87.40a	2.25b
90	100	196.27	85.60ab	2.29b
120	100	195.77	84.90ab	2.30b
150	100	197.82	82.87b	2.38a
Environment				
Comfort	250	200.64a	86.07	2.33a
Heat stress	250	192.18b	84.78	2.26b
SEM		8.58	3.91	0.10
*p*-value				
Zn		0.9868	0.0386	0.0060
Environment		0.0007	0.1491	0.0061
Zn × environment		0.5180	0.4134	0.4630
Regression				
Linear		0.9542	0.0078^1^	0.0007^2^
Quadratic		0.6166	0.3897	0.0746

Means followed by different letters in the same column differ significantly according to the *t*-test (*p* < 0.05). SEM, standard error means; *n*, number of observations per treatment group.

1 WG_1–21 days_ = −0.0348*x* + 88.654; (*R*^2^ = 0.86).

2 FCR_1–21 days_ = 0.0009*x* + 2.217; (*R*^2^ = 0.74).

The thermal environment where the birds were housed affected the FI and FC. Under thermal comfort, the birds exhibited higher FI (*p* = 0.0007) and FCR (*p* = 0.0061) compared to the heat stress environment. WG, in turn, was not influenced by the thermal environment to which the animals were subjected (Table 3). Heat stress promoted a reduction in FI but did not promote an equivalent reduction in WG, a response that improved FCR in the referred environment.

Over the total rearing period, from 1 to 42 days of age, an effect of the interaction (*p* = 0.0019) between Zn levels and thermal environment was observed on WG (Table 4).

**Table 4:** Performance of Japanese quail aged 1 to 42 days old, supplemented with zinc levels in the diet and reared under different thermal environments (*n* = 500).

Zn levels (mg kg^−1^)	*n*	Feed intake (g bird^−1^)	Weight gain (g bird^−1^)	Feed conversion ratio (g/g/bird)
30	100	540.37	131.72	4.10
60	100	518.83	132.11	3.93
90	100	527.83	128.88	4.09
120	100	527.50	128.85	4.10
150	100	518.57	126.22	4.11
Environment				
Comfort	250	532.82	133.22a	4.00b
Heat stress	250	521.25	125.73b	4.15a
SEM		34.21	4.71	0.65
*p*-value				
Zn		0.1033	0.8625	0.2634
Environment		0.0609	<0.0001	0.0133
Zn × environment		0.7369	0.0019	0.6209
Regression				
Linear		0.0708	0.1442	0.2773
Quadratic		0.6277	0.1916	0.3648

Means followed by different letters in the same column differ significantly according to the *t*-test (*p* < 0.05). SEM, standard error means; *n*, number of observations per treatment group.

There was effect of the thermal environment on FCR; quail maintained under heat stress had a 3.75% increase in FCR compared to those raised under thermal comfort (Table 4). It was observed that, despite the lack of an effect from the environment on FI, there was a reduction in quail WG and an increase in FCR, which may be attributed to the inefficient utilization of the nutrients offered by the feed, significantly hindering quail performance.

**Figure 1:**
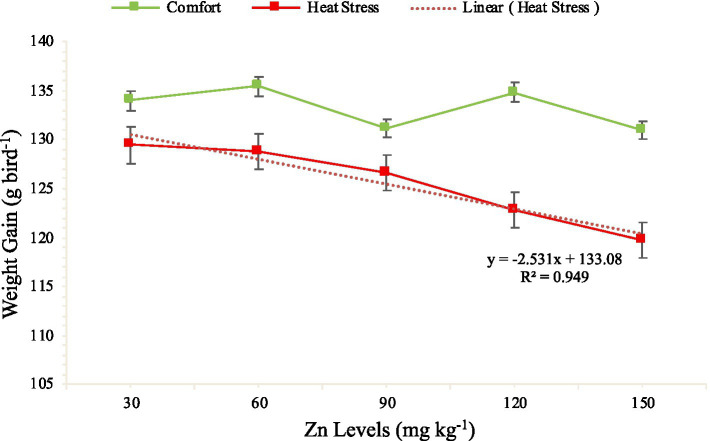
Breakdown of the interaction effect between zinc levels and thermal environments on the weight gain of quail aged 1 to 42 days.

The effect of the interaction between the dietary Zn levels and the thermal environment on WG are presented on Figure 1. The Zn levels exhibited a decreasing linear effect (*p* < 0.0001) on the WG of quail maintained under heat stress; however, for the quail maintained under thermal comfort, the Zn levels had an increasing linear effect (*p* = 0.0997) on WG.

Based on the results, it was observed that Zn levels above 30 mg kg^−1^ of feed did not have beneficial effects on the WG of the quail maintained under heat stress.

### 3.2 Organ and carcass weight

There was no interaction between the dietary Zn levels and the thermal environment on the organ and carcass weight in the initial phase rearing phase (Table 5). According to the regression analysis, the spleen and cloacal bursa were not influenced by Zn levels.

**Table 5:** Organ and carcass weight of Japanese quail at 21 days of age, supplemented with zinc levels in the diet and reared under different thermal environments (*n* = 100).

Zn levels (mg kg^−1^)	n	Weight (g)			
		Spleen	Cloacal bursa	Liver	Carcass
30	20	0.059	0.156	1.937ab	58.69
60	20	0.059	0.156	2.055a	59.09
90	20	0.064	0.153	1.964ab	57.43
120	20	0.057	0.150	1.958ab	57.35
150	20	0.059	0.156	1.870b	55.31
Environment					
Comfort	50	0.0638	0.168a	1.967	55.91
Heat stress	50	0.0561	0.141b	1.905	51.76
SEM		0.020	0.048	0.205	13.556
*p*-value					
Zn		0.7547	0.9898	0.0283^1^	0.5050
Environment		0.0564	0.0007	0.0547	0.0581
Zn × environment		0.4880	0.4824	0.2845	0.4274
Regression					
Linear		0.8520	0.8985	0.0681	0.7662
Quadratic		0.4422	0.7837	0.0292	0.2267

Means followed by different letters in the same column differ significantly according to the *t*-test (*p* < 0.05). SEM, standard error means; *n*, number of observations per treatment group.

1 Liver weight_21 days_ = −4 × 10^−5^ × 2 + 0.0064*x* + 1.7457; (*R*^2^ = 0.79).

The effect of dietary Zn levels was observed for the liver. Regression analysis of Zn levels revealed that liver weight showed a quadratic response (*p* = 0.0292), and it was estimated that 80 mg of dietary Zn is required to achieve maximum weight. It is assumed that use of Zn amounts above 80 mg kg^−1^ in diets to quail, even without reaching toxic levels, reduces the metabolic activity of the liver, reducing, thus, its weight.

The environment influenced the weights of all organs and of the carcass. The quail that were maintained under heat stress had a weight reduction of 13.72% for the spleen, 19.15% for the cloacal bursa, 5.56% for the liver and 2.70% for the carcass compared to quail that were maintained under a thermal comfort environment (Table 5). This weight reduction may be related to the oxidative damage that heat stress causes to the cell membranes of immune organs.

**Figure 2:**
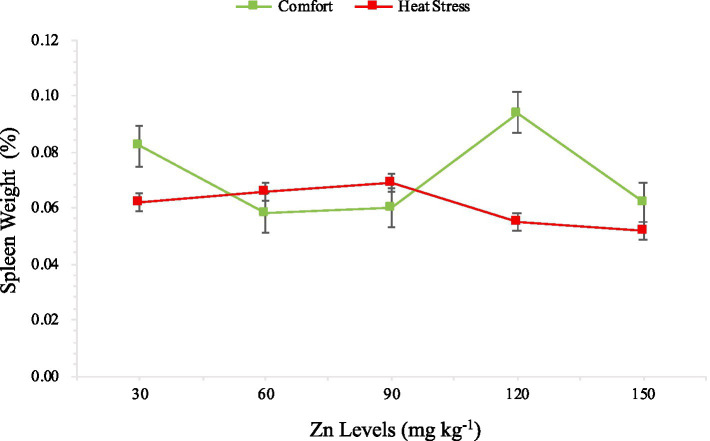
Breakdown of the interaction effect between zinc levels and thermal environments on spleen weight of quail at 42 days of age.

At 42 days, there was an interaction between Zn levels and the thermal environment only for the spleen weight (Table 6 and Figure 2).

**Table 6:** Organ and carcass weight of Japanese quails at 42 days of age, supplemented with zinc levels in the diet and reared under different thermal environments (*n* = 100).

Zn levels (mg kg^−1^)	*n*	Weight (g)			
		Spleen	Cloacal bursa	Liver	Carcass
30	20	0.072	0.141	3.23	85.38
60	20	0.062	0.134	3.19	86.30
90	20	0.064	0.125	3.23	87.38
120	20	0.075	0.138	3.17	88.40
150	20	0.057	0.139	3.16	87.92
Environment					
Comfort	50	0.071	0.146a	3.45a	89.41a
Heat stress	50	0.069	0.124b	2.96b	84.59b
SEM		0.034	0.058	0.564	5.378
*p*-value					
Zn		0.1829	0.8021	0.9827	0.3824
Environment		0.0525	0.0378	<0.0001	<0.0001
Zn × environment		0.0243	0.9034	0.3660	0.1013
Regression					
Linear		0.3563	0.8806	0.5879	0.0742
Quadratic		0.7492	0.3092	0.8827	0.4242

Means followed by different letters in the same column differ significantly according to the *t*-test (*p* < 0.05). SEM, standard error means; *n*, number of observations per treatment group.

### 3.3 Tibia breaking strength

There was no significant effect on the interaction between environment and dietary zinc levels for the bone strength parameter. However, the higher level of zinc inclusion in the diet provided a better resistance response of the tibia of Japanese quails at 42 days of evaluation (Table 7).

**Table 7:** Bone strength (BS) of the tibia of Japanese quails at 42 days of age, supplemented with zinc levels in the diet and in different thermal environments (*n* = 100).

Zn levels (mg kg^−1^)	*n*	Bone strength (kgf)
30	20	2.546b
60	20	3.079ab
90	20	3.105ab
120	20	3.178ab
150	20	3.372a
Environment		
Comfort	50	3.254a
Heat stress	50	2.857b
SEM		0.67
*p*-value		
Zn		0.0235
Environment		0.0117
Zn × environment		0.6591
Regression		
Linear		0.0008^1^
Quadratic		0.2943

Means followed by different letters in the same column differ significantly according to the *t*-test (*p* < 0.05). SEM, standard error means; *n*, number of observations per treatment group.

1 BS_42 days_ = 0.0061*x* + 2.4872; (*R*^2^ = 0.89).

Likewise, the environment characterized as thermal comfort favored a better resistance response of the tibia of these animals.

## 4 Discussion

### 4.1 Zootechnical performance

The results of this study demonstrated that Zn supplementation levels and thermal environment significantly affect the zootechnical performance of Japanese quails, aligning with previous findings in the literature. During the initial phase (1 to 21 days), higher Zn supplementation levels did not result in superior performance compared to lower levels. This suggests that the lower Zn levels were sufficient to meet the birds’ requirements during this stage, without the need for additional supplementation. This conclusion is consistent with the findings of Lopes et al. (11), who reported a reduction in the productive efficiency index in broiler chickens subjected to diets with 120 mg of Zn kg^−1^, indicating that elevated Zn levels may impair performance in the initial growth phase.

Moreover, previous studies have shown no significant interaction between different Zn sources and the thermal environment on zootechnical performance during early rearing stages (12). In the present study, the thermal environment directly influenced feed intake (FI) and feed conversion ratio (FCR), while weight gain (WG) was not affected, suggesting that the environment alone does not uniformly modify all performance variables.

The reduction in FI observed in birds under heat stress, as reported both in this study and in previous research, reflects a common physiological adaptation in homeothermic animals exposed to high temperatures, as described by Berto (9). This reduction in feed intake aims to minimize metabolic heat production, leading to a lower FCR, albeit with potential impacts on weight gain. In contrast, birds kept under thermal comfort exhibited higher FI and FCR, highlighting the direct influence of the environment on feeding behavior and feed efficiency.

During the total rearing period (1 to 42 days), the significant interaction between Zn levels and thermal environment highlighted that high Zn levels were not beneficial under heat stress conditions. Indeed, quails kept under heat stress exhibited a linear reduction in WG with increasing Zn supplementation, whereas those kept under thermal comfort showed a tendency for increased WG. This suggests that Zn utilization efficiency is modulated by the environment, emphasizing the need for nutritional strategies tailored to thermal conditions.

These findings hold important practical implications. High Zn levels, particularly those exceeding 30 mg/kg, may be detrimental to birds under heat stress, as also reported in previous studies (11). It is therefore advisable to adjust supplementation levels according to the thermal environment to optimize performance without compromising feed efficiency. Under thermal comfort, the birds’ optimized metabolic capacity can support higher Zn levels, whereas moderate levels are sufficient and preferable under heat stress conditions.

Finally, the results of this study reinforce that nutritional adjustments, particularly for microminerals such as Zn, should be based on a detailed understanding of the interaction between diet and environment. Beyond improving zootechnical performance, such strategies can minimize the impacts of heat stress, promoting greater efficiency and sustainability in intensive production systems.

### 4.2 Organ and carcass weight

The results of this study demonstrated that high temperatures caused significant reductions in organ and carcass weights in quail, corroborating the findings of Piray and Foroutanifar (13), who observed significant reductions in the relative weights of immune organs in broilers subjected to heat stress. These data reflect the impact of the thermal environment on bird metabolism, especially under heat conditions, where thermoregulatory adaptation mechanisms prioritize heat dissipation at the expense of growth and immune function.

The liver, as one of the most metabolically active organs, was particularly affected. A quadratic response to dietary Zn levels was observed, with the maximum weight estimated at 80 mg Zn kg^−1^. This suggests that higher levels of Zn may not be metabolically utilized, resulting in a reduction in liver weight. Additionally, heat stress promoted a decrease in liver weight, likely linked to the metabolic modulation observed under high temperatures, where internal activities are adjusted to minimize metabolic heat production, as similarly reported in studies with broilers.

Reductions in spleen and cloacal bursa weights under heat stress highlight the susceptibility of immune organs to oxidative damage. These structures play critical roles in immune response, and the impact of heat can severely compromise the birds’ immunocompetence. Interestingly, previous studies have reported increases in cloacal bursa weight in broilers receiving dietary Zn (14); however, this effect was not observed in the quail in this study, suggesting potential species-specific differences or variations in Zn response under different environmental conditions.

The loss of carcass weight in birds maintained under heat stress was another notable finding. This result may be attributed to increased respiratory rate, an essential thermoregulatory mechanism in birds. Panting, although effective for heat dissipation, requires greater thoracic muscle activity, increasing glycogen and adenosine triphosphate (ATP) consumption, as noted by Ezzati et al. (15). This process can compromise the development of breast muscle, the primary muscle mass in birds, thereby reducing total carcass weight.

The interaction observed between Zn levels and the thermal environment on spleen weight at the end of the rearing period reflects the complex relationships between nutrition and environment in modulating the immune performance of birds. While Zn appears to play a more effective role in maintaining immune homeostasis under thermal comfort conditions, its effects are more limited under heat stress, likely due to increased metabolic demands and oxidative damage.

These findings emphasize the need for nutritional strategies that consider environmental conditions to optimize bird health and performance. Adjusting dietary Zn levels, combined with measures to mitigate heat stress, could be an effective approach to enhancing bird resilience in intensive production systems.

### 4.3 Tibia breaking strength

The results of this study demonstrate the significant role of zinc (Zn) in bone metabolism, particularly in its interaction with calcium (Ca) during bone synthesis. Zinc acts as a cofactor and structural component of carbonic anhydrase, which is crucial for providing carbonate ions necessary for both eggshell formation and bone mineralization (16). This function underscores the observed positive impact of dietary Zn levels on tibia breaking strength in Japanese quail. These findings align with Sahraei et al. (17), who reported that supplementation with 150 mg/kg of Zn improved tibia strength in growing broilers, and Kumar et al. (18), who observed that higher dietary Zn concentrations promoted greater Zn deposition in the tibia, enhancing bone strength.

The quail in this study were in the initial laying phase, during which Zn demands for eggshell formation and bone synthesis are particularly high. The results suggest that higher dietary Zn levels ensured sufficient carbonate ion availability for these processes, leading to stronger tibias.

The thermal environment also significantly influenced tibia strength, with quail housed in thermal comfort conditions displaying stronger tibias than those in a heat-stress environment. Heat stress induces physiological changes such as increased respiratory rate, which aims to lower body temperature but often results in acid–base imbalance and respiratory alkalosis due to excessive CO_2_ elimination (19). This condition disrupts the animal’s electrolyte balance, which is critical for Ca metabolism. During respiratory alkalosis, a substantial proportion of Ca becomes complexed with blood proteins, reducing the availability of free ionized Ca for bone deposition, ultimately compromising bone strength (19).

The interaction between dietary Zn and Ca is vital for bone quality, but the effects of heat stress demonstrate that even optimal nutrient levels cannot fully mitigate the adverse impacts of environmental stressors. These results highlight the need for integrated management strategies combining nutritional supplementation with measures to alleviate heat stress. Such strategies would optimize both welfare and production outcomes by maintaining bone health and minimizing losses associated with heat stress.

## 5 Conclusion

The results of this study indicate an antagonistic relationship between high levels of zinc (Zn) in the diet and the zootechnical performance of quails, where the addition of the highest level of Zn (150 mg kg^−1^) impaired performance. Supplementation with the available Zn from the basal diet (30 mg kg^−1^) was sufficient to ensure satisfactory weight gain, better feed conversion, and appropriate carcass and liver weights of Japanese quails in the initial phase of rearing.

On the other hand, at 42 days of age, supplementation with 150 mg kg^−1^ of Zn provided greater resistance to tibial breakage in quails, regardless of thermal conditions. This reinforces the importance of adjusting Zn supplementation according to the needs of quails at different stages of rearing.

Thus, the study highlights the need for a balanced nutritional approach, considering both adequate Zn levels and the management of thermal stress. The combination of appropriate Zn levels in the diet and environmental management, especially regarding thermal stress, are crucial to optimize productive performance, bone health, and the well-being of quails. The nutritional Zn requirements for quails in the initial phase of rearing may be lower than previously established, without compromising performance. Gradual Zn supplementation according to the needs of the production stage and environmental conditions is key to ensuring the health and productivity of the birds.

## Data Availability

The raw data supporting the conclusions of this article will be made available by the authors, without undue reservation.
